# Be Prepared for a Mental Illness “Pandemic” in China: Too Early to Celebrate the Victory Over COVID-19

**DOI:** 10.1017/dmp.2021.11

**Published:** 2021-01-11

**Authors:** Kaimin Li, Yan Wu, Kefeng Li

**Affiliations:** 1 Department of Hematology, The Affiliated Yantai Yuhuangding Hospital of Qingdao University, Yantai, Shandong, China; 2 Department of Clinical Laboratory, The Affiliated Yantai Yuhuangding Hospital of Qingdao University, Yantai, Shandong, China; 3 School of Medicine, University of California, San Diego, San Diego, California, USA

**Keywords:** post-COVID-19, society reopen, mental health, pandemic response

## To the Editor

Since April 2020, China lifted its lockdown on Wuhan, the epicenter of the coronavirus disease 2019 (COVID-19) pandemic. The authorities are now celebrating the success over COVID-19 and focusing on economic recovery. Many psychological assistance measures established during the pandemic have been terminated. In fact, lessons from severe acute respiratory syndrome (SARS) outbreak in 2003 had taught us that the pandemic could have profound and potentially long-term impacts on mental health.^1^ The deleterious consequences of COVID-19 might be even worse than those caused by SARS in 17 y ago due to the new problems that China is currently facing after the outbreak.^2^ Additionally, nationwide fears rise over a second coronavirus wave in China. All these factors could exacerbate the adverse effects of COVID-19 on mental health.

Because mental health interventions have not been integrated into emergency response plans, we urge policy-makers in China to approach the mental illness “pandemic” ahead of time through the following steps, including psychological aid (A), medication stockpile (S), substance abuse prevention (P), and financial relief (R) (ASPR).

## Psychological Aid (A)

It is necessary to establish a long-term mechanism for mental health assistance to the public, at-risk groups, and health-care professionals. Operational planning guidelines are urgently needed, which contain ethical principles, screening protocols, and types of intervention to various groups. Because China has a severe shortage of psychiatrists (1.49 psychiatrists/100,000 population),^3^ trained medical students can provide psychological first aid to the public.

## Medication Stockpile (S)

It is time to increase the national stockpile for common and essential psychiatric medicines, ensure the supply security, and prevent price gouging. The COVID-19 pandemic will encourage the funding agencies, and pharmaceutical vendors to enhance their Research and Development (R&D) investments in developing new psychiatric drugs.

## Illicit Drug and Alcohol Abuse Prevention (P)

Alcohol abuse or dependence frequently coexists with various psychiatric disorders.^4^ The authorities could consider preventing excessive alcohol use through evidence-based community strategies. Law enforcement departments should expand the surveillance and punishment of illegal drug trade.

## Financial Relief (R)

Financial stress is the leading cause of mental illnesses.^5^ In an effort to boost domestic consumption after the COVID-19 pandemic, the authorities have distributed electronic consumption coupons among the local residents through third-party payment platforms, such as Alipay and WeChat Pay by means of smartphone. However, this approach has limited help for people who have already had little money to spend or do not have a smartphone, and who are not familiar with the way to receive electronic coupons. The government should consider more powerful rescue strategies to these people, such as cash handout, which has been done in many countries such as the United States, Japan, and Canada.

The impact of the COVID-19 pandemic on mental health can occur in the immediate aftermath and then persist over long periods. The Chinese government must address public mental health needs by developing and implementing well-coordinated preparedness and response plans. The time for action is now.
